# An experimental model to investigate the targeting accuracy of MR-guided focused ultrasound ablation in liver

**DOI:** 10.1186/1479-5876-12-12

**Published:** 2014-01-16

**Authors:** Lorena Petrusca, Magalie Viallon, Romain Breguet, Sylvain Terraz, Gibran Manasseh, Vincent Auboiroux, Thomas Goget, Loredana Baboi, Patrick Gross, K Michael Sekins, Christoph D Becker, Rares Salomir

**Affiliations:** 1 Faculty of Medicine, University of Geneva, Geneva, Switzerland; 2 Department of Radiology, University Hospitals of Geneva, Geneva, Switzerland; 3 Clinatec/LETI/CEA, Grenoble, France; 4 Siemens AG, HealthCare Sector, Erlangen, Germany; 5 Siemens Ultrasound, Montainview, CA, USA

**Keywords:** MR guided HIFU, Ballistic marker, Tracer encapsulation, Targeting, Pre-clinical quality assurance

## Abstract

**Background:**

Magnetic Resonance-guided High Intensity Focused Ultrasound (MRgHIFU) is a hybrid technology that aims to offer non-invasive thermal ablation of targeted tumors or other pathological tissues. Acoustic aberrations and non-linear wave propagating effects may shift the focal point significantly away from the prescribed (or, theoretical) position. It is therefore mandatory to evaluate the spatial accuracy of ablation for a given HIFU protocol and/or device. We describe here a method for producing a user-defined ballistic target as an absolute reference marker for MRgHIFU ablations.

**Methods:**

The investigated method is based on trapping a mixture of MR contrast agent and histology stain using radiofrequency (RF) ablation causing cell death and coagulation. A dedicated RF-electrode was used for the marker fixation as follows: a RF coagulation (4 W, 15 seconds) and injection of the mixture followed by a second RF coagulation. As a result, the contrast agent/stain is encapsulated in the intercellular space. Ultrasonography imaging was performed during the procedure, while high resolution T1w 3D VIBE MR acquisition was used right after to identify the position of the ballistic marker and hence the target tissue. For some cases, after the marker fixation procedure, HIFU volumetric ablations were produced by a phased-array HIFU platform. First *ex vivo* experiments were followed by *in vivo* investigation on four rabbits in thigh muscle and six pigs in liver, with follow-up at Day 7.

**Results:**

At the end of the procedure, no ultrasound indication of the marker’s presence could be observed, while it was clearly visible under MR and could be conveniently used to prescribe the HIFU ablation, centered on the so-created target. The marker was identified at Day 7 after treatment, immediately after animal sacrifice, after 3 weeks of post-mortem formalin fixation and during histology analysis. Its size ranged between 2.5 and 4 mm.

**Conclusions:**

Experimental validation of this new ballistic marker method was performed for liver MRgHIFU ablation, free of any side effects (*e.g.* no edema around the marker, no infection, no bleeding). The study suggests that the absolute reference marker had ultrasound conspicuity below the detection threshold, was irreversible, MR-compatible and MR-detectable, while also being a well-established histology staining technique.

## Introduction

High Intensity Focused Ultrasound (HIFU) is fundamentally a propagating wave capable of producing localized thermal lesions [1-3]. A large variety of US transducers, either single element or phased array, with continuous or pulsed-mode [4], have been described [5-7] for this non-invasive approach, proving the growing interest in the scientific and medical communities.

The highest temperature elevation induced by the HIFU beam is expected to occur around the focal point, but every penetrated tissue in the pathway of the wave will also be heated at various levels depending on acoustic and physiological properties and on its relative position from the transducer. Cavitation, boiling or an increased absorption coefficient in the tissue may induce a thermal drift of the ablative lesion towards the transducer [8]. Far field of the transducer can also be affected by thermal heating, especially if a reflective interface is present [9]. Refraction on pre-focal interfaces [10], spatial misregistration of the device or geometrical distortion of MR guidance images may also yield lateral offsets of the effective position of the focal point.

Magnetic resonance guided HIFU (MRgHIFU), in particular using near real time Proton Resonance Frequency Shift (PRFS) thermometry (MRT), is at present the only FDA approved HIFU technology [11]. This approach has been clinically investigated to treat malignant tumors using prototype or off-label devices originally dedicated to uterine fibroid ablation [12-20].

Theoretically, there are no limitations on the lesion size treatable by MRgHIFU [21]. Sequential MRgHIFU elementary sonications were evaluated clinically [20], indicating the technical feasibility despite the long treatment times [12-22]. Volumetric MRgHIFU ablation has also been investigated, demonstrating that a large and uniform ablation zone can be rapidly obtained using continuous sonication and mechanical displacement of the transducer [23,24], interleaved electronic and mechanical displacement of the focus [25] or fully electronic steering of the beam [6]. Moreover, studies using ultrasound guidance proposed different methods to increase the lesion size, such as: inertial cavitation that significantly enhanced the heat deposition at the HIFU focus [26,27], focus splitting for larger coagulations regions [28] or using a multifocal thoroidal transducer that produced composite lesions [29,30].

Effective spatial control of an induced thermal lesion during rapid volumetric ablation should be considered a major safety issue [9]. Together with the capacity to create large, rapidly achieved lesions, any volumetric sonication paradigm must also guarantee accurate spatial control of the HIFU ablative lesion, with predictable position of the ‘gravity’ center of the ablative region. To our best knowledge, available reports are missing providing experimental data to support that volumetric thermal ablations are indeed centered on a specific predefined target in 3D, i.e. a histological “gold standard” [31].

Different studies describing ballistic markers for HIFU ablations have already been published. Melodelima *et al*[32,33] proposed a tumor-mimicking model made by injecting a warm solution at 65°C that polymerizes in hepatic tissue and forms a typically 1 cm size discrete lesion that was detectable by ultrasound imaging and gross pathology. That model was a large unperfused 1 cm hyperechoic lesion on sonograms (US reflecting) and the polymerized solution had attenuation coefficient of 0.39 dB.cm^−1^ at 1 MHz, as compared to 0.68 dB cm^-1^ for liver parenchyma [34]. Recently, Pichardo *et al*[35] proposed using the same agarose-based solution for MR targeting and thermal monitoring during HIFU sonication on living static tissue (rabbit muscle). The model applied to static muscular tissue in rabbits induced significant edema probably due to intrinsic temperature of the injected solution, while the animals were sacrificed immediately post-treatment. Therefore, the final dimensions of the lesion and its spatial relation to the ballistic model were not assessed histologically.

In order to improve and/or to assess spatial control of ablation, we present here an experimental method to create a user-defined ballistic target that can be used as an absolute reference marker prior to any HIFU trials. The marker must be irreversible, MR compatible and detectable both in pre-operative MRI and in post-mortem histology and has to enable the evaluation of the spatial control achieved during MR-guided HIFU ablation of liver lesions, in particular concerning the lesion’s ‘center of gravity’. At the end of the procedure, the contrast agent/stain must be stably encapsulated in the intercellular space, where perfusion, diffusion and/or metabolism are limited or suppressed.

## Material and methods

### Principle and instrumentation

The marker fixation paradigm consisted of three steps: 1. interstitial RF coagulation of a small region of tissue, 2. injection at the same location of 0.1 mL of a mixture of methylene blue (Patent Blue V Sodium 2.5%, Guerbet SA, France) doped with 1% gadolinium-DTPA (Dotarem, Guerbet, France), and 3. a second RF coagulation, identical to the first. The role of RF coagulation is to trap the substance in the tissue by immediately ceasing metabolism and capillary circulation.

**Figure 1:**
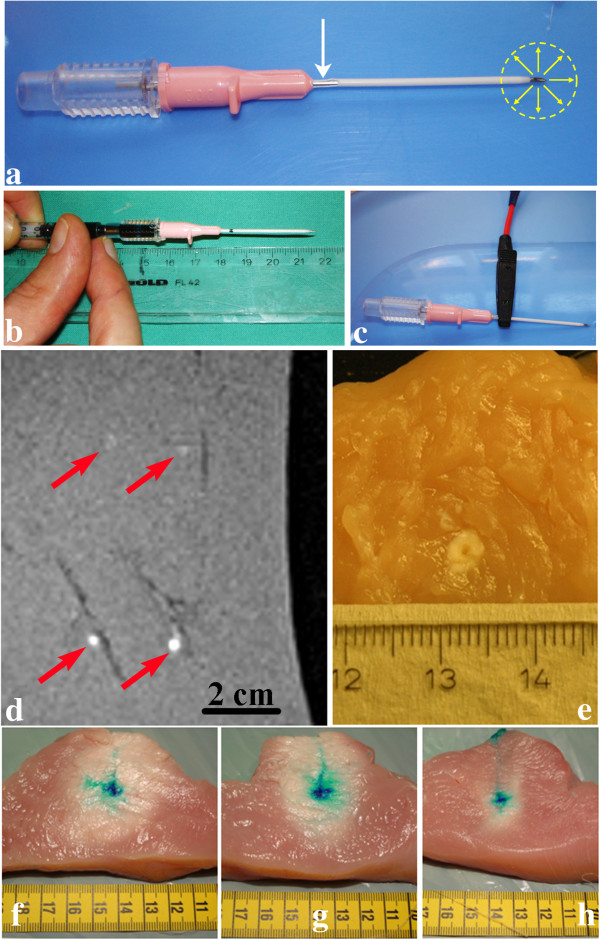
**The dedicated RF needle and *ex vivo *marker results. a-c)** Monopolar electrode engineered to affix the histological marker. Note the hemicylindrical proximal cut of the PFTE isolator sheath (arrow) and electrical clamp at this site. Deployment of the electrical field around the tip is also illustrated. **d)**. *Ex-vivo* T1w image (VIBE 3D isotropic sequence) of four independent thermal lesions (see red arrows) obtained post-RF ablation. The upper lesions correspond to a coagulated spherical area created without injection of the Gd-doped staining and the lower lesions were obtained after standard fixation of the marker, with injection of Gd-doped methylene blue. **e)**. Macroscopic slice showing the optimal RF thermal lesion, visible as a white spheroidal volume of colorless tissue (non-injected procedure). **f) - h)**. *Ex vivo* macroscopic slices of volumetric MRgHIFU ablation, cut perpendicular to the focal plane, showing the coagulative lesions successfully centered on the ballistic target (high intensity blue spot in three different slices). The prescribed patterns of sonication were 7 x 2 foci, 6 x 2 foci and 3 x 2 foci, respectively, 4 mm inter-foci gap, with a delivered energy of 18 kJ, 15.4 kJ and 7.7 kJ, respectively.

A dedicated RF electrode was used for local coagulation and contrast substances injection. This was designed to operate in monopolar mode and was manufactured based on 1. a percutaneous entry thin wall needle 18 G/7 cm (Cook Medical, Bloomington, IN), or 2. radiopaque *i.v.* catheter 16G/14 cm, (Abbocath-T, Hospira, Inc, Lake Forest, IL). A square 6 cm^2^ copper sheet in contact with the skin (*via* saline serum) was used as ground contact (see Figure 1a-c for details). The catheter is sterile, low cost and individually packaged in a blister bag, and is for single use only, as is the resulting home-made RF electrode. The puncture needle is covered by a polytetrafluoroethylene (PTFE) sheath, which is an excellent electrical insulator (volume resistivity > 10^18^ Ωm [36]). The sheath covers the full length of the puncture needle, excepting its tip (1.2 mm). This PTFE sheath will isolate the tissue from the electric field, which will deploy only at the tip of the puncture needle, thus creating a sharply localized thermal ablation. Moreover, a semi-circular 5 mm long cut was performed at the proximal edge of the PTFE sheath, where a conductive clamp made the electrical contact between the puncture needle and the (+) contact of the RF clinical generator (Celon*POWER*, Celon AG, Teltow, Germany) working at 475 kHz [37].

To calibrate the power and duration for optimal fixation and visualization of the ballistic model, RF thermal lesions with various parameters were created with the catheter-based electrode in *ex vivo* turkey white meat (degassed during 30 minutes to remove any exogenous gas bubbles). The lesions were imaged by MR and further visually examined after the meat sample was sliced.

### HIFU system

*Ex vivo* and *in vivo* MRgHIFU experiments were conducted with two separate systems. The first system (#1) used a randomized 256 element phased array transducer (Imasonic, Besançon, France) operating in the frequency range from 974 to 1049 kHz, maximum acoustic power 300 W, with a natural focal length and aperture of R = 130 mm and D = 140 mm, respectively. Each active element of the transducer is a disk of 6 mm diameter. The HIFU platform uses a programmable 256 channel generator and a 2D positioning mechanism in the horizontal (XZ) plane (both from Image Guided Therapy, Pessac, France). Each channel delivers an RF sine wave, with independent control of the phase, frequency and amplitude. Electronic steering of the focal point was available within a -3 dB revolution ellipsoid of axes 30 mm, 30 mm and 50 mm around the natural focus. The update of phases and amplitudes for the 256 channels takes less than 10 ms and the focal point position could be changed 20 times per second to follow any complex trajectory. The positioning mechanism for the transducer provides two independent horizontal translation axes, i.e. X and Z in the magnet frame, within a displacement range of +/- 80 mm in each direction with 0.5 mm accuracy. In-house written software packages for treatment planning and hardware control were plugged in the real-time kernel of the graphical user interface (Thermoguide™, Image Guided Therapy, Pessac, France). Online temperature map displays were provided by the software interface.

The second HIFU system (#2) used was a research prototype abdominal HIFU system operating at 1 MHz and comprising a high density therapeutic array (18 432 elements in a 13 cm × 11 cm rectangular acoustic aperture, element size 0.5 × 1 mm^2^, Siemens Healthcare, Ultrasound Division, Montainview, CA [38,39]. This array was capable of high acoustic energy flux per unit area (>12 Wac/cm^2^ average, >25 Wac/cm^2^ peak; >2 kWac total average aperture power), significant electronic steering (55 degrees in azimuth and 45 degrees in elevation, no mechanical displacement) and also rapidly scanned focal beam patterns (>1 kHz beam focal point rate). This array was the acoustic power delivery component in an integrated software controlled MR guided HIFU system mated to a Siemens 3 T Trio MRI scanner.

### MR imaging

A 3 T whole body MRI scanner (Magnetom Trio a Tim system, Siemens AG, Germany, maximum gradient strength = 45 mT/m and maximum slew rate = 200 T/m/s) was used in the presented study for planning and HIFU temperature monitoring purposes. A high resolution T1w 3D gradient-echo (VIBE) acquisition (TE/TR/TA/FA/BW =1.6 ms, 4 ms, 2.55 min, 10°, 650 Hz/Px) was used to predefine the position of the ballistic target in the investigated zone, far from bone and other adjacent structures. The spatial resolution and scan time were, respectively 0.8 × 0.8 × 0.8 mm^3^/2 min 55 s in static tissue and 1.2 × 1.2 × 1.2 mm^3^/50 s in liver under forced apnea. On-line multi-planar high-resolution PRFS-based MR thermometry [40-42] was used to monitor the HIFU ablation in real time. Temperature elevation was monitored using a segmented GRE-EPI sequence [43] with EPI factor = 11, FOV = 128 mm square (*ex vivo*) or 200 mm square (*in vivo*), voxel size = 1 × 1 × 5 mm (*ex vivo*) or 1.56 × 1.56 × 5 mm (*in vivo*), 3 interleaved slices, TR/TE/FA = 50 ms to 70 ms/9 ms/15°, bandwith = 738 Hz/pixels, temporal resolution 2.5 to 3.5 s. A thermal dose was calculated using the Sapareto model [44]. The HIFU beam propagated through the aperture of, respectively, a receive-only 11 cm diameter loop coil (for *ex vivo* and rabbit investigations) positioned in a horizontal plane, or a dedicated 3-element interventional coil (Clinical MR Solutions, Brookfield, WI, for liver interventions) wrapped around the animal.

### *In vivo* protocol

All animal procedures were approved by the Geneva University Institutional Animal Care and Use Committee and by the Cantonal Veterinary Authority of Geneva. Blood oxygen saturation, body temperature and exhaled CO_2_ were monitored continuously.

Four rabbits (3–4 kg, New Zealand, females) were premedicated with ketamine (30 mg/kg; Pfizer, Zürich, Switzerland) and midazolam (0.2 mg/kg; Roche Pharma, Reinach, Switzerland). After placing an intravenous access using an ear vein, the rabbits were intubated and mechanically ventilated (Servo Ventilator 900D, Siemens-Elema, Sweden) using 6-m long ventilation tubing and anesthesia was maintained by continuous intravenous administration of 1% propofol (perfusion rate 20 ml/h, Astra Zeneca AG, Zug, Switzerland) delivered by a distant non MR-compatible clinical syringe pump (B-D Pilot A; Becton Dickinson Infusion Systems, Brezins, France). The breathing rate was approximately 40 breaths/min. In addition, to prevent any contraction of the leg during MRgHIFU ablation, the sciatic nerve of the investigated limb was locally anesthetized with 1 ml of 1% lidocaine (Bichsel AG, Interlaken, Switzerland), as well as an *i.v.* injection of pancuronium (0.2 mg/kg; Essex Chemie AG, Lucerne, Switzerland) administered 2 minutes before the MRgHIFU ablation. For MRgHIFU treatment in the thigh muscle, the rabbit was positioned in lateral decubitus. The HIFU system #1 produced the volumetric ablation theoretically prescribed, to be centered on the created ballistic target. One marker was produced in each animal followed by volumetric HIFU ablation. The main parameters of HIFU ablation in rabbit thigh were: 150 Wac power, 120 sec duration, duty-cycle nearly 100%, 2 × 5 foci pattern, 4 mm inter-foci gap.

A similar protocol for ballistic marker investigation was performed in liver in six pigs. Two markers/liver were produced. In two animals, sonication using MRgHIFU system #2 and PRFS MR thermometry were performed under respiratory gating using the optical signal generated by an in-house built opto-mechanical sensor [45] attached to the animal’s abdomen. The breathing rate was approximately 10 breaths/min. Each animal was positioned prone for sub-xiphoid acoustic access to the liver parenchyma. Sonication parameters were 450 Wac power, 60 sec duration, duty-cycle around 50%, 5 mm diameter disk-like scanning pattern. A single marker was targeted in each treated animal. Note that acoustic power values are determined from radiation force calibration prior to MRgHIFU experiments.

After marker fixation (see below) and HIFU ablations (if any), the animals were awakened and followed up for 7 days. Postoperative analgesia was administered (2 × 0.01 mg/kg/day Buprenorphine, Essex Chemie AG, Lucerne, Switzerland) during the follow up period. At day 7, the sonicated region (thigh or liver) was imaged with Gd-enhanced T1-weighted MRI and afterwards the animal was sacrificed. The investigated organ was removed post-mortem and fixed in 4% formaldehyde. This was further processed for macroscopic analysis and microscopic histology. The evaluation of complication was based on imaging follow-up and necropsy findings.

### Interventional procedure

After local aseptic treatment of the skin, the home-built monopolar RF electrode was inserted in the animal to create a marker under US guidance outside the MR magnet bore. An ultrasonography scanner (Antares, Siemens Medical Solutions, Mountain view, CA, USA), equipped with an abdominal CH4.1 phased array imaging head was used for both B-mode (frequency range 1.54 to 3.6 MHz) and second harmonic imaging (receive frequency range between 4 and 4.4 MHz).

The *in vivo* procedure was performed in a sterile manner by 2 experienced interventional radiologists outside the MR scanner room with the animal lying supine on the table. The skin was incised with a scalpel and the 16 G electrode was inserted under real time US guidance (see equipment above). The electrode was introduced into the middle of the thigh or in the right hepatic lobe. For ablation, an average of 4 W was delivered during 15 seconds for each impact (before and after a staining injection). At the end of RF power application, a hyperechoic area typically 1 cm in diameter on US monitoring was clearly visible around the electrode tip. This effect was due to boiling cavitation bubbles [43], and was used as a per-operatory indicator of sufficient local ablation around the electrode tip. Note, if the hyperechoic cloud of bubbles was not visible, the RF ablation was repeated with gradually increased power up to 10 W. The needle was then withdrawn without ablating the needle track until it reached the hepatic capsule, where a second marker was generated to obtain a superficial tag, facilitating post-mortem identification of the treated hepatic lobe.

The acoustic properties of the affixed marker were investigated using the same US imager during the RF ablation and post-RF ablation. The described ballistic model was further used to mimic a ‘virtual’ tumor-center in order to assess the spatial control of HIFU ablation *ex vivo* and *in vivo* (sheep and pigs, respectively) on static and moving tissue. MRI was used interactively to identify the affixed marker, and hence the volume of tissue to be targeted, using a T1w 3D gradient-echo (VIBE) sequence (see above). The 3D information was then used to position the transducer and to calculate the HIFU beam forming so as to align the focal point with the marker.

The spatial control of ablation achieved was evaluated 7 days later by comparing the pre-defined position of the imitated tumor ballistic target and the position of necrotic and fibrotic areas (i.e. the ‘gravity center’) identified in contrast-enhanced T1w sequences. Finally, on post-mortem histology the center of the ablative lesion was compared to the local marker i.e. fixed methylene blue staining.

### Macroscopic analysis and histology

For macroscopic analysis, the investigated organ was excised and formalin fixed for 3 weeks. Post-fixation, the specimen was encapsulated in dual-component polyurethane foam formed into a cubic shape to enable mechanical slicing. The entire cube obtained after a 15 min polymerization period was sliced in 1–1.2 mm thick slices using a commercially available slicer (Gemma 300, SIRMAN s.p.a., Pieve di Curtarolo, Italy). Optical scans (600 to 2400 dpi) of each individual macroscopic slice were systematically recorded. Co-registration was further improved using in-house written Matlab code (The Mathworks Inc, US), if needed, and stack tiff-type images were generated for further integration and 3D post-processing using Osirix Software (Osirix Foundation, Geneva, Switzerland). The chosen macroscopic slices of interest were quickly investigated on a microscope (HES staining, Mictotome Leica RM 2135, slice thickness: 5 μm) or frozen at -80°C for further investigation on a CM3050 cryostat.

## Results

The optimal parameters for marker fixation and MR detection were determined to be 2 W RF power during 30 sec, resulting in 60 J energy deposition, with a 18 G electrode, and 4 W RF power during 15 sec, also resulting in 60 J energy deposition, with a 16 G electrode. A higher power level was in general suboptimal as it triggered impedance cutoff and subsequently smaller lesions. Figure 1d shows T1-weighted MR images obtained *ex vivo* after monopolar RF ablation using the home-built electrode, with and without local injection of the mixture of methylene blue and Gadolinium chelate. The marker created by localized energy deposition and fixation of the contrast agent is clearly visible under MR and can be conveniently used to pre-define the zone to be ablated by HIFU, centered on the so-created target. The size of the histological marker ranged between 2.5 and 4 mm (Figure 1e). To illustrate the marker’s usefulness in assessing the spatial accuracy of the thermal lesion, we present in Figure 1f-h three independent examples of *ex vivo* thermal lesions obtained by volumetric MRgHIFU (see the whitened ellipsoidal area inside the fresh *ex vivo* sample), centered on the ballistic target (high intensity blue spot) within 2 mm spatial accuracy. Some spreading of the methylene blue staining along the muscle fibers was unavoidable when slicing the fresh meat across the central plane of the lesion. However, the color contrast between the native marker and the spread staining is evident.

**Figure 2:**
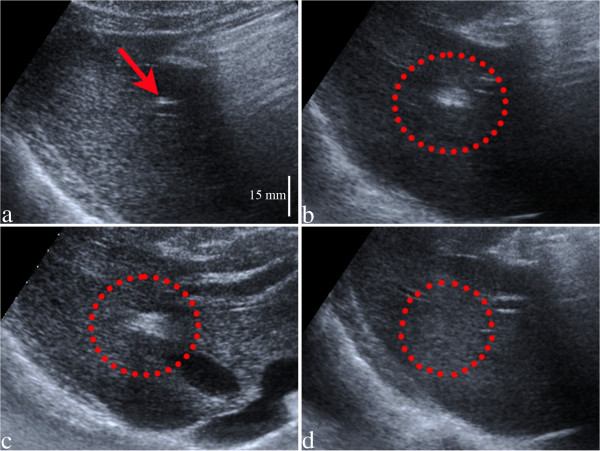
**US images (harmonic imaging) before (a), during (b, c) and after (d) RF ablation in pig liver (please find embedded scale distance).** The electrode alone is visible during the planning period, before RF heating (**a**, see the red arrow). During RF ablation, the gas bubbles created around the electrode are clearly seen in the early **(b)** and late **(c)** stages of the procedure. No gas bubbles are visible one minute after the end point of RF ablation due to wash-out with perfusion **(d)**.

During the *in vivo* interventional procedure of marker fixation, the ultrasonography clearly visualized the tip of the RF electrode before ablation (Figure 2a), and the boiling cavitation during RF ablation (Figure 2b,c). The precise position of the induced ablation could be chosen before RF coagulation, based on initial images where the RF electrode tip is visible. This confirmation in real time is very useful, making it possible to change the needle’s position to the preferred point and then fixate the marker. The occurrence of the “white cavitation” cloud of bubbles is a direct indication that RF ablation was effective. The “wash-out” of the cloud bubbles required approximately one minute after the end-point of RF coagulation, as seen in Figure 2d. After the bubbles wash out, no ultrasound indication of the marker’s presence could be observed, with standard (retro-diffused, or back-scattered) ultrasound imaging performed in the frequency range 2-4 MHz, although the experiments were conducted in optimal conditions *ex vivo* where the marker was positioned a few centimeters only from the imaging transducer. The imaging wavelength at 4 MHz being approximately 10 times smaller than the marker itself, missing of any marker signature cannot be attributed to acoustic diffraction. Therefore this is interpreted as no significant change in the acoustic impedance in tissue because of the marker, within the experimental resolution. This evidence is not covering the thermal effects involving the absorption of the HIFU beam on the marker, therefore specific MRgHIFU experiments were conducted, as described in Appendix.

**Figure 3:**
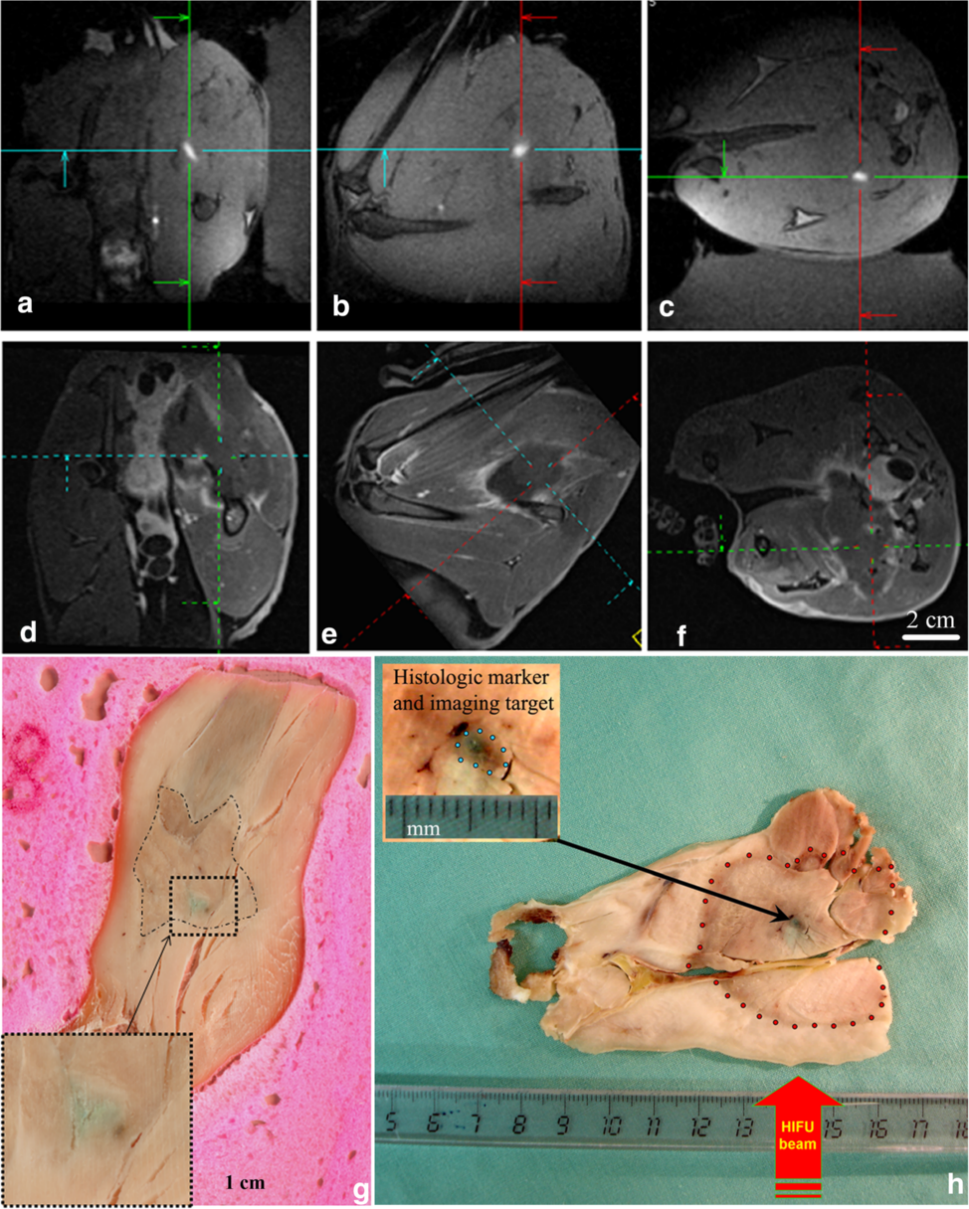
***In vivo *ballistic marker in rabbit thigh followed by HIFU sonication. a) - c)**: T1w 3D (VIBE) images of the ballistic target model, shown in situ just before MRgHIFU sonication. The sagittal (feet first), coronal and axial planes are shown from left to right, respectively. The RF-affixed marker appears as a bright spot. **d) - f)**: Gd-enhanced T1w 3D (VIBE) images 7 days post-treatment of the same animal, showing the necrotic and fibrotic lesion. The multi planar reconstruction crossing point indicates the approximate position of the pre-defined ballistic target model. **g)** Macroscopic findings corresponding to the same rabbit treatment (3 weeks formalin fixation, followed by toluene bath dehydration and 8 months conservation). The piece was sliced along the sagittal plane (head first) and the dashed contour delineates the ablative lesion. The methylene blue staining is seen to be misaligned with the center of the actual MRgHIFU thermal lesion by approximately 1 cm. Coagulation necrosis showed a complete loss of striation (down in the corner, zoomed view). **h)** Macroscopic illustration of another rabbit thigh sliced after 3 weeks post mortem formalin fixation. The MRgHIFU volumetric lesion (delineated manually by dots) is here perfectly centered on the ballistic target model, the latter visible as a dark stained spot.

Figure 3 displays *in vivo* MR T1-weighted images pre- and 7-days post-marker fixation and HIFU therapy in static tissue, in rabbit thigh. The ballistic target is clearly seen prior to the HIFU treatment in each section through the 3D reconstruction (a-c). The apoptotic and necrotic area formed 7 days after treatment is visible as a hypo intense signal zone surrounded by a hyper intense rim (d-f) and corresponds to the planned HIFU ablation. Methylene blue marker trapped in tissue by thermo coagulation was clearly detected in different animals after either 3 weeks (Figure 3h) or 8 months (Figure 3g) of post-mortem formalin fixation. Frame g is showing an eccentric lesion with respect to the ballistic marker as a result of per-operatory HIFU-induced muscle contraction, detected on line during the treatment (data not shown). Frame h is illustrating a thermal lesion precisely centered on the ballistic marker when intra-operatory tissue motion did not occur. The size of the final HIFU ablations (mean values measured on 4 rabbits), measured at Day 7, after Gd injection, were: in plane long axis = 30 mm, short axis = 21 mm and height (parallel to the HIFU beam) = 45 mm.

**Figure 4:**
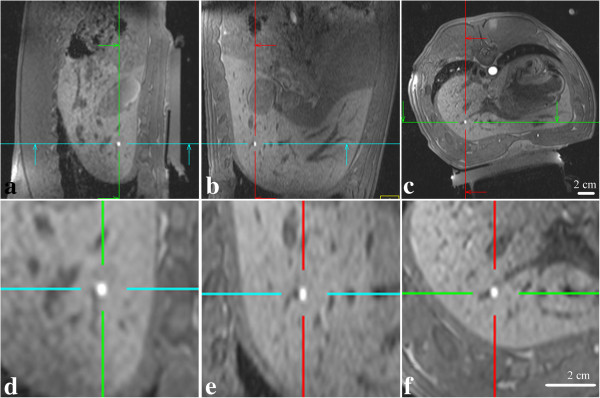
**T1w 3D (VIBE) images of the ballistic target model obtained in a pig liver, 10 minutes after its fixation (shown FOV is 270 mm square for a) - c) and 90 mm square for d) - f)).** Note the strong T1 contrast of the target, easily detectable in all slices: sagittal **(a, d)**, coronal **(b, e)** and axial **(c, f)**. Zoomed images (x3) are shown in frames **d) - f)**.

**Figure 5:**
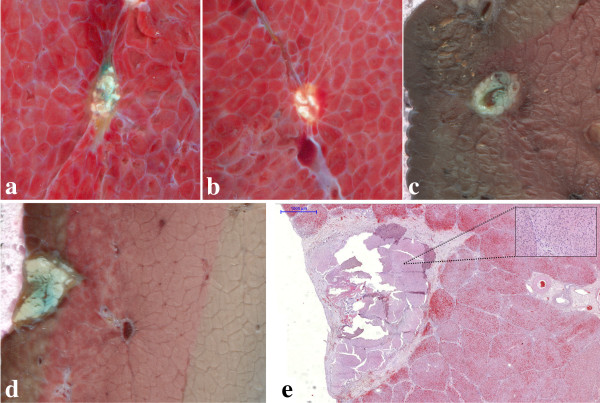
**Post-mortem findings of different ballistic markers created in pig liver. a)**, **b)** The liver was freshly cut at Day 7, while the marker was created either with injected methylene blue **(a)** or without methylene blue injection **(b)**. **c)**, **d)** Macroscopic illustration of the ballistic marker after 3 weeks of formalin fixation. The RF lesions correspond to spheroid areas of ablation surrounded by healthy tissue in frames **a) -c)**. The RF ablation shown in frame **d)** was performed at the liver periphery and corresponds exactly with the microscopic histology, illustrated in frame **e)** (see embedded distance scale). The FOV shown in frames **a) - c)** is 15 × 15 mm and in frame **d)** is 15 × 20 mm.

The ballistic target model was easily detectable *in vivo* in pig liver, as a strong hyper-signal in T1w 3D VIBE images (Figure 4) immediately after fixation, due to the trapped Gd-based contrast agent. The small volume of RF coagulated tissue and the trapped tracers induced no measurable magnetic perturbation in the phase maps acquired with the PRFS temperature-sensitive GRE-EPI sequence during HIFU sonication. This behavior enables the direct use of reference-free PRFS-calculated temperature maps [46,47] without any correction specific to the marker. Seven days after the marker procedure, the thermal lesions induced by RF ablation could be identified in freshly cut liver samples (Figure 5a,b). Macroscopic slices indicated a blue-to-green colored volume of coagulated tissue (frame a) for the methylene blue stained ballistic marker and a whiter zone (frame b) for a case without methylene blue. No methylene blue was detected outside the RF-ablated tissue, indicating normal clearance of the un-trapped staining. Post-mortem macroscopic illustration (frames c and d) and microscopic histology (frame e, same lesion as shown in frame d) of the ballistic marker (inside the lobe – c, and at the periphery of the lobe - d and e) after 3 weeks of formalin fixation clearly indicates the ablated zone surrounded by healthy tissue. Histopathology indicated a complete disorganization of hepatocytes and the absence of red cells due to the sinusoids destruction in the center of the marker. More peripherically, intra-cellular edema was found. The lesion was sharply delineated by a few hundred microns thick fibro-inflammatory rim. The rest of the hepatic parenchyma had a normal aspect, with clear delineation of lobuli and no visible side effects due to the interventional procedure. Please note that no HIFU sonication was performed in the examples illustrated in Figures 4 and 5. For *in vivo* studies, the marker was imaged (T1w VIBE MR sequence) in situ at 10 and 80 minutes after RF procedure, and no alteration was detected between these 2 time points.

**Figure 6:**
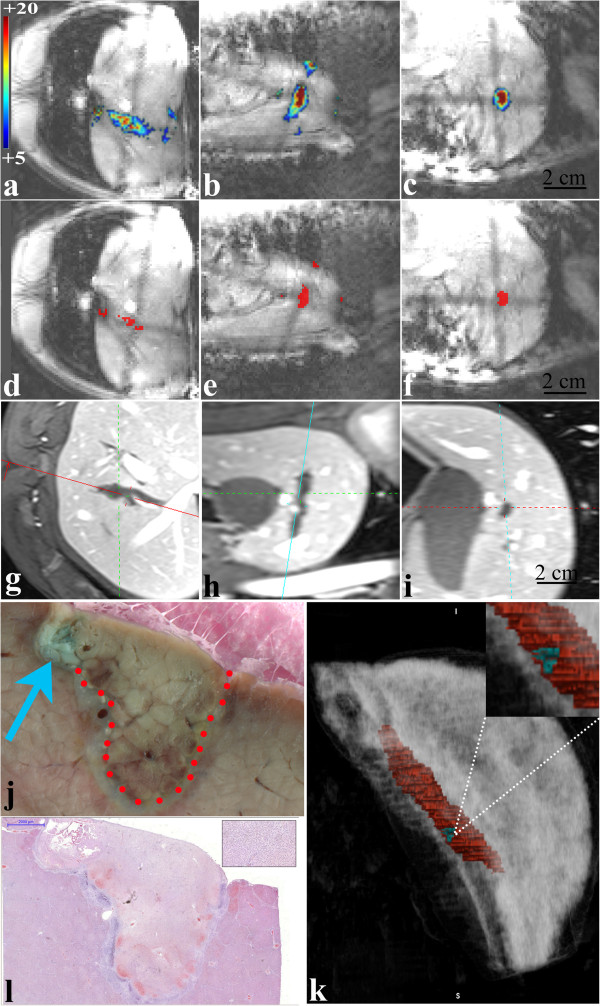
***In vivo *ballistic marker in pig liver followed by HIFU sonication.** Temperature elevation (°C) **(a-c)** and lethal thermal dose (>240CEM43) maps **(d-f)** obtained at the end of HIFU sonication in pig liver. **g) - i)** T1w VIBE images of the final lesion (HIFU and RF ablation) in pig liver, at Day 7 after Gd administration. **j)** Post-mortem macroscopic illustration of the ballistic marker and HIFU ablation after 3 weeks of formalin fixation. The difference between the 2 lesions is visible due to the blue coloration of the ballistic marker. FOV = 20 × 20 mm. **k)** 3D reconstruction of the liver lobe containing the HIFU lesion and the ballistic marker illustrated in frame **j**: entire HIFU lesion (elongated shape, in red) and the RF marker (in blue) at the periphery; **l)** microscopic histology, same slice as shown in frame **j)**.

An *in vivo* ballistic marker followed by HIFU ablation in pig liver is shown in Figure 6. Temperature (a-c) and thermal dose maps (d-f) in three different planes indicate the thermal build-up accumulation at the end of the HIFU sonication. Seven days after the treatment, after Gd administration, the final lesion (g-i) could be easily identified in T1w VIBE images. Moreover, the lesion was also identified in post-mortem macroscopic (j) and microscopic analysis (l), having a similar ablation shape. In frame j the two ablated regions are easily distinguished: the blue arrow indicates the RF coagulated marker (identified by the presence of methylene blue), positioned peripheral to the HIFU ablation, delimited by a dotted red line. Microscopic histology (frame l) revealed a clear difference between the ablated zone and healthy tissue, with normal cells showing clear striation by hematoxilin-eosin staining, as well as peripheral nuclei, while areas of coagulation necrosis appeared with lighter staining and showing a complete loss of lobular structure. A 3D reconstruction (Osirix Software) of the hepatic lobe containing the final lesion (same as in frame j) is shown in frame k, indicating the HIFU lesion and the RF marker at the periphery. The shortest distance between the centers of the two lesions was 5 mm. The calculated volume of the HIFU lesion was 1.935 cm^3^, while the volume of the created marker was 0.0235 cm^3^ in the presented case.

## Discussion

We describe here a new method to create a user-defined ballistic target that can be used as an absolute reference marker, based on trapping a mixture of gadolinium chelate and methylene blue (hystology stain) and using sharply localized RF energy deposition, causing immediate cell death and coagulation. The gadolinium-based contrast agent was easily detectable by pre-operative MR imaging; therefore, this method provided a minimally invasive and robust ballistic target, mimicking a small ‘virtual’ tumor, and can be conveniently used as a target for MRgHIFU ablations. According to pathologists, the methylene-blue does not affect the microscopic histology staining, while it is trapped within the coagulated tissue. The large majority of pre-clinical and clinical studies using MR-guided HIFU technology are reporting temperature elevation below 70°C, since approaching the boiling regime is likely to produce local disruption of the magnetic susceptibility [9,43] and thus errors in PRFS MR thermometry. A sharp RF coagulation is required whenever the HIFU treatment does not result in immediate necrosis, because the HIFU-induced apoptosis in the treated area does not prevent wash out of the staining until all metabolism ceases. We demonstrated that methylene blue was trapped by immediate coagulation of the tissue using localized RF energy deposition, and that any un-trapped staining was cleared all around, including from inside the HIFU-ablated tissue. Staining was detectable immediately after animal sacrifice (see Figure 5), after 3 weeks of formalin fixation (see Figure 6) and also after eight months of formalin fixation (see Figure 3). This demonstrates a highly stable histology marker, compatible with semi-chronic follow up protocols, where the living tissue may deform during the time the animal is allowed to survive after sonication. No local or systemic complications related to the marker were noticed in 10 animals during the survey period of 7 days. The minimally invasive interventional procedure observing sterile conditions should be at low risk.

During RF ablation, boiling bubbles in the tissue were easily detected with ultrasonography, during approximately one minute after the end-point of RF ablation in pig liver (see Figure 2). Beyond this transient period, the created target did not modify the local echogenicity of tissue in the range 1.57 - 4.4 MHz, nor the local magnetic susceptibility (neutral versus PRFS thermometry) and induced no observable side effects in tissue. The marker involves tissue coagulation and injection of a small volume of stable liquid state solutions, while its size is comparable to the wavelength of HIFU. These conditions yield the minimal acoustic perturbation of HIFU beam – if any – by a marker that is conveniently visualized with conventional resolution T1-weigthed MR sequences. If no HIFU ablation of tissues surrounding the marker is performed, the small coagulative volume of the marker itself is very likely to be reabsorbed by the tissue in the long term. Due to real time US visualization of the electrode tip while the operator manipulates the needle towards the investigated zone, a major advantage of this ballistic marker is the ability to choose the precise position of the induced ablation. If the needle is not at the right place, the operator can alter the position until the optimal position is reached before delivering the RF energy. Another advantage of this model can be considered the possibility of creating multiple markers in the same organ, due to the time effective procedure and to their point-like aspect. The RF electrode can be repositioned in the organ, or removed completely, changed and inserted again in the body using another entry point. The effectiveness of ablation is confirmed with US detection of white cavitation, while staining is confirmed by MR imaging of trapped Gd from the same mixture.

Correct evaluation of what tissue is ablated by HIFU sonication and precise determination of HIFU-induced lesion position versus the treatment planning cannot be done if the animal is sacrificed immediately post-treatment. Therefore, for our study, the animals were followed up for 7 days. Histological analyses both macro- and microscopic clearly distinguished the two types of lesions (ballistic marker and HIFU ablation). This can indicate, post-treatment, whether the HIFU lesion was centered on the target or not. Moreover, various prefocal thermo-acoustic effects (like thermal drift induced by cavitation or differential absorption in the traversed tissues) may be evidenced with this approach.

Accurate targeting in abdominal organs during breathing is a challenging problem. Unlike previous reports dealing with static conditions during the sonication [32,35], our sonications in pig liver were performed under continuous breathing and gating. Internal organ drift between targeting and sonication steps [48], acoustic aberration of the HIFU beam versus the theoretical beam forming in homogeneous tissue and geometric distortion of MR images sequence-specific are inherent limits to the achievable accuracy for centering the lesions on the T1-contrast marker. Here, a larger population for *in vivo* quantitative assessment of the targeting accuracy with the available MRgHIFU device was not defined as a goal.

The presented method demonstrated no risk of tissue contamination with air bubbles and no irregular heating pattern was observed on PRFS temperature maps during HIFU sonication, as opposite to [35]. Importantly, the needle and the syringe were mounted together and filled with liquid solution prior to the incision. The interventional procedure to fix the marker here was safe and successfully performed in all cases within approximately 15 minutes. No specific training was requested for the interventional radiologist. The very low volume of injected liquid was delivered at room temperature and no physical or chemical transformation will occur spontaneously after some critical period of time. Thus, there is no constrained timing for executing the interventional procedure. This is particularly important to allow the interventionist sufficient time to accurately perform the image guided positioning of the electrode. Tissue effraction is minimal and direct assessment of the ballistic accuracy of millimeter-range (i.e. elementary) HIFU lesions aiming to overlap the marker location is meaningful due to the small size of the marker itself, unlike the previous model [32,35].

There are different applications where the presented marker model may be suitable. The presented ballistic model is expected to be a convenient procedure to validate the accuracy of HIFU ablation in any organ, static or mobile. Beside tissue-dependent or device-dependent targeting errors, the entire problem of motion tracking errors in moving targets can further tackled.

Other applications of our affixed marker may involve minimally invasive tissue ‘tattooing’ as landmarks for diseased tissue/tumor or healthy structures at risk to be delineated for surgery, for instance “missing metastasis”. Current preoperative marking techniques to guide radiotherapy or surgery include guide wire locators (with the associated risks of migration, transection and scheduling conflicts), radioactive injections [49,50] and charcoal suspensions [51], which are invasive. Radiotherapy is often guided using external tattoos and internal clips in tumor beds or at biopsy sites. Substituting the methylene blue staining with a radio-opaque agent (*e.g.* iodine-based) may render the marker visible to X-Ray techniques.

Multi-modal imaging contrast agent can also be considered for ablative trapping using the methodology described in this paper (*e.g.* correlation of PET with MR images). The coagulating energy applied here was radio frequency, but laser ablation devices (LITT) may also be used with an appropriate design of the delivery tool.

## Conclusion

HIFU is fundamentally a propagating wave with a limited precision of a-priori prediction of the focus position in tissue during sonication. We presented here an experimental method to investigate the targeting accuracy of MR-guided focused ultrasound ablation, by fixing a ballistic target detectable with both pre-operative MR and post-mortem histology. The marker’s ultrasound conspicuity was below the detection threshold on a clinical ultrasound imager. The marker was established irreversibly, induced no changes in local magnetic susceptibility and its size was comparable to the HIFU wavelength. It showed no side effects in tissue and can be considered as a long-term stable histology staining method.

## Appendix

### Investigation of marker’s influence on elementary sonication induced thermal build-up

**Figure 7:**
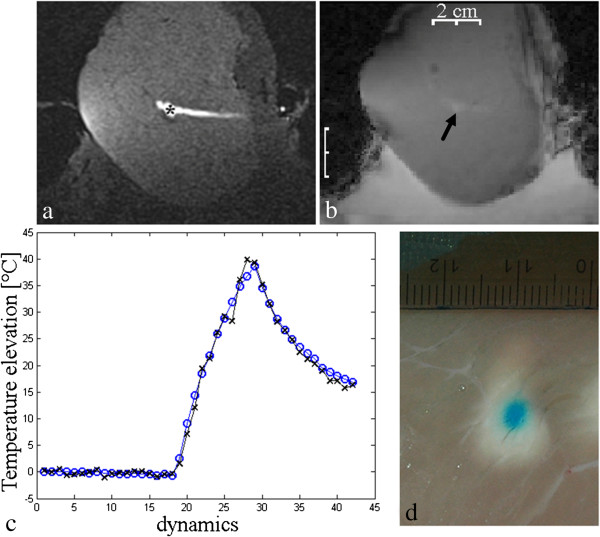
**Appendix.** Example of precise targeting of a marker with elementary HIFU sonication: T1-w VIBE **(a)** and in situ magnitude GRE-EPI thermometry **(b)** images showing the marker and the trajectory of the RF needle; temperature elevation **(c)** during HIFU sonication on a marker (blue plot) and in bulk tissue away from the marker (black plot) and a camera picture **(d)** of the elementary HIFU lesion centered on the ballistic marker.

Specific experiments were designed and performed, consisting of targeting the marker with single focus elementary HIFU sonication while guaranteeing high targeting precision, as both the marker and the elementary lesion were small. To achieve high ballistic precision, we have labeled with MR contrast agent and visual staining not only the marker but also the trajectory of the RF needle serving as electrode (Figure 7a). Moreover we have checked the marker’s position not only on the planning 3D image but also on the magnitude of GRE-EPI thermometry images in situ (Figure 7b), controlling that a low energy pilot sonication yielded PRFS phase shift at the correct location prior to high energy ablation (108 W, 30 s). An example of accurate targeting of a marker with elementary sonication is provided in Figure 7d. Under these strictly controlled conditions, on four repeated experiments in two samples of *ex vivo* Turkey breast muscle, we could not measure any significant influence of the marker on the MR temperature elevation versus heating bulk tissue away of the marker, see Figure 7c. For best robustness, the thermal comparison on vs. out of marker was performed with mechanical 2D translation of the transducer and invariant electronic beam forming. Considering the effective volume of the RF ablated tissue produced with the marker and, respectively, the effective volume of an elementary HIFU ablation, it appears macroscopically that the presence of the marker has not a measurable influence on the thermal build up.

## Competing interests

The authors declare that they have no competing interests.

## Authors’ contributions

Manuscript definition of intellectual content: LP, ST, PG, CDB, RS; Manuscript preparation/revision/review: LP, MV, ST, RS; *in vivo* protocol design and implementation: LP, MV, RB, ST, GM, TG, LB, RS; data acquisition, analysis and interpretation: LP, RB, GM, RS; design and manufacturing of the specific instrumentation for the study: VA, PG, KMS, RS; literature research: LP, MV; competitive funds raising: ST, CDB, RS; guarantee of integrity of entire study: PG, CDB, RS. All authors read and approved the final manuscript.
